# Supplementary material for: Inhibition of iRhom1 by CD44-targeting nanocarrier for improved cancer immunochemotherapy
Source: Nat Commun. 2024 Jan 4;15:255. doi: 10.1038/s41467-023-44572-6 (PMC10766965; doi:10.1038/s41467-023-44572-6)
Supplement: Supplementary file 1 — Supplementary Information [file 41467_2023_44572_MOESM1_ESM.pdf]

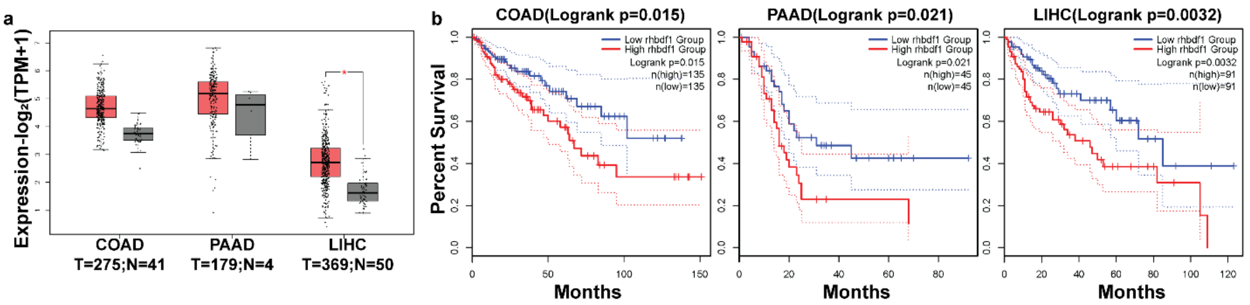

Supplementary Figure 1. Analysis of TCGA data by GEPIA2 online tool. P-value = 0.01 in LIHC group. **(a)** iRhom1 is overexpressed in several types of cancers. **(b)** High expression levels of iRhom1 are associated with poorer clinical prognosis. COAD: colon adenocarcinoma; PAAD: pancreatic adenocarcinoma; LIHC: liver hepatocellular carcinoma. Data are presented as mean ± s.d. in a. Statistical analysis was performed by two-tailed Student's t-test for comparison in a and log rank test for comparison in b.

a

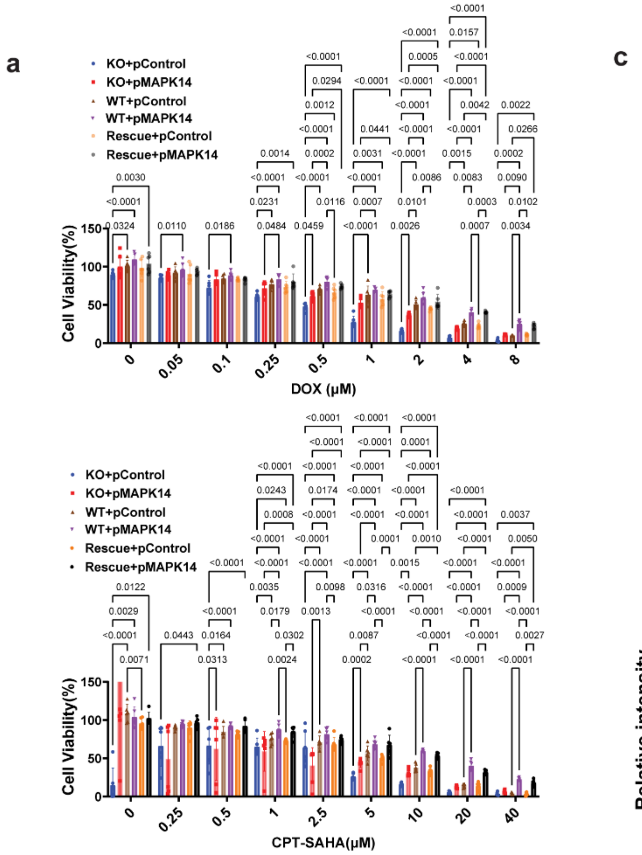

IC50 (μM)	KO +pControl	KO +pMAPK14	WT +pControl	WT +pMAPK14	Rescue +pControl	Rescue +pMAPK14
DOX	0.854	1.98	3.87	6.96	3.11	5.96
CPT-SAHA	2.51	5.05	8.09	19.13	7.65	16.86

b

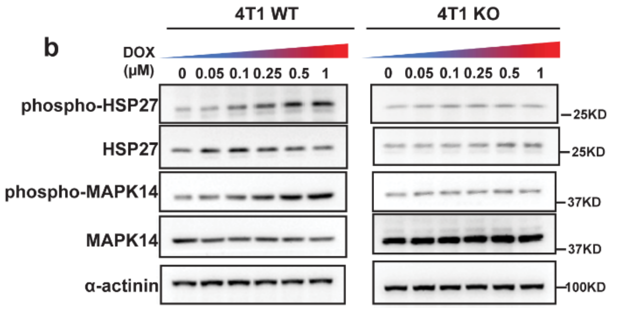

d

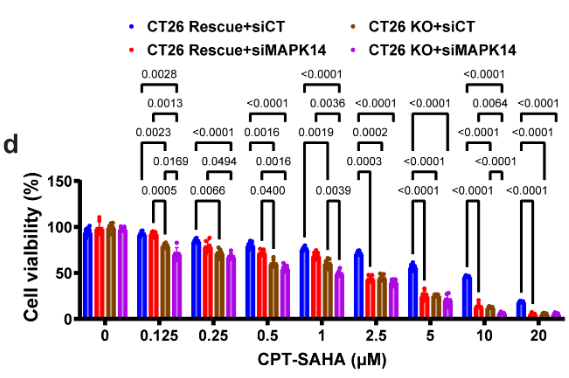

c

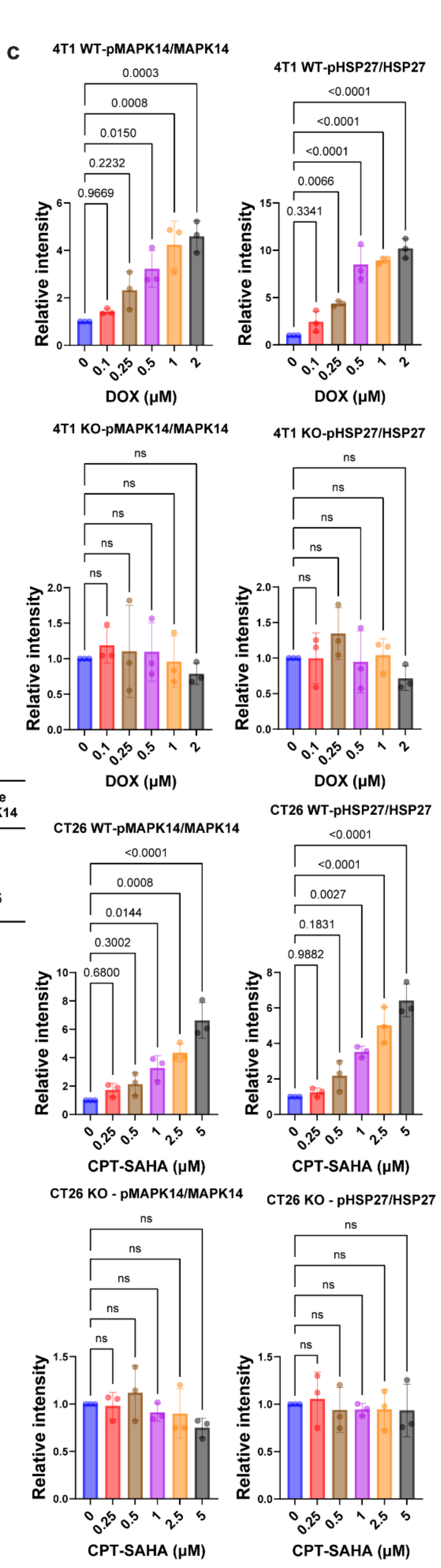

Supplementary Figure 2. (a) MTT cytotoxicity assay of CPT-SAHA or DOX treatment on iRhom1 WT cells, iRhom1 KO cells, iRhom1 KO cells with re-expression of MAPK14, and iRhom WT cells with over-expression of MAPK14, and the corresponding IC_{50}. N=6 independent samples. **(b)** Changes in the protein levels of MAPK14-HSP27 axis after treatment with various concentrations of DOX in 4T1 iRhom1 KO cells and WT cells. **(c)** Densitometry quantification of the induction of MAPK-HSP27 axis in WT/iRhom1 KO cells following treatments with various doses of DOX or CPT-SAHA. N=3 independent experiment. **(d)** MTT cytotoxicity assay of treatments with various concentrations of CPT-SAHA in CT26 iRhom1 KO cells or CT26 iRhom1 KO cells with iRhom1 re-expression (rescue), with or without knockdown of MAPK14 respectively. N=6 independent samples. Data are presented as mean ± s.e.m. in a, c, d. Statistical analysis was performed by one-way ANOVA with Tukey's post hoc test for comparison in a, c and d. Data are representative of two independent experiments in a, d, and three independent experiments in b, c. Source data are provided as a Source Data file for all panels.

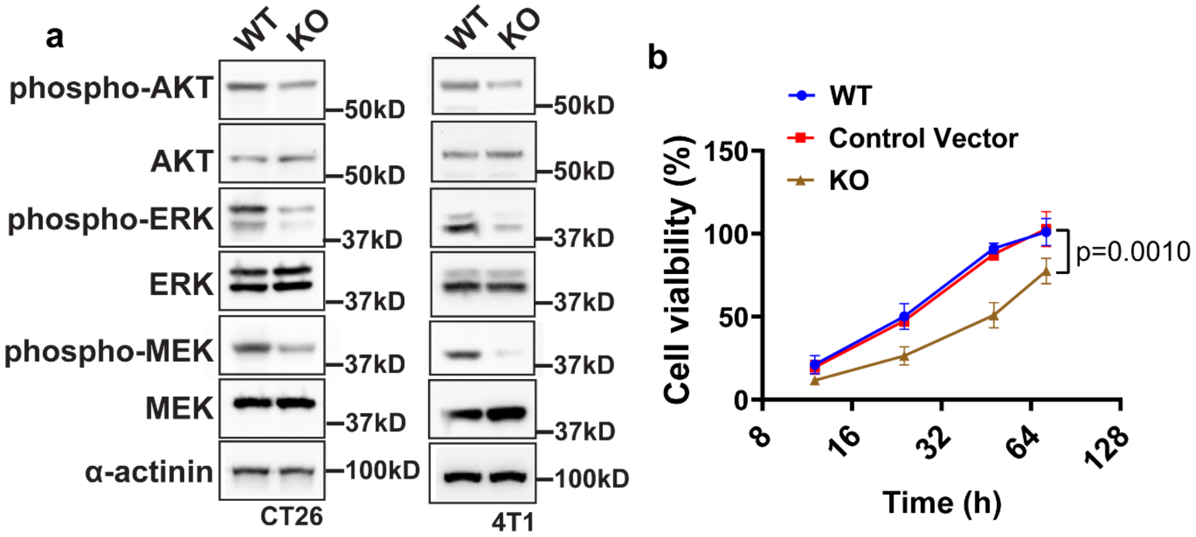

Supplementary Figure 3. (a) Changes in the protein levels of several pro-survival kinases (phosphorylated) in iRhom1 KO cells compared to WT cells. **(b)** MTT cell viability assay of CT26 WT, control vector and iRhom1 KO cells after different culture time. N=6 independent samples. Data are presented as mean ± s.e.m. in b. Statistical analysis was performed by one-way ANOVA with Tukey's post hoc test for comparison in b. Data are representative of two independent experiments in a, b. Source data are provided as a Source Data file for all panels.

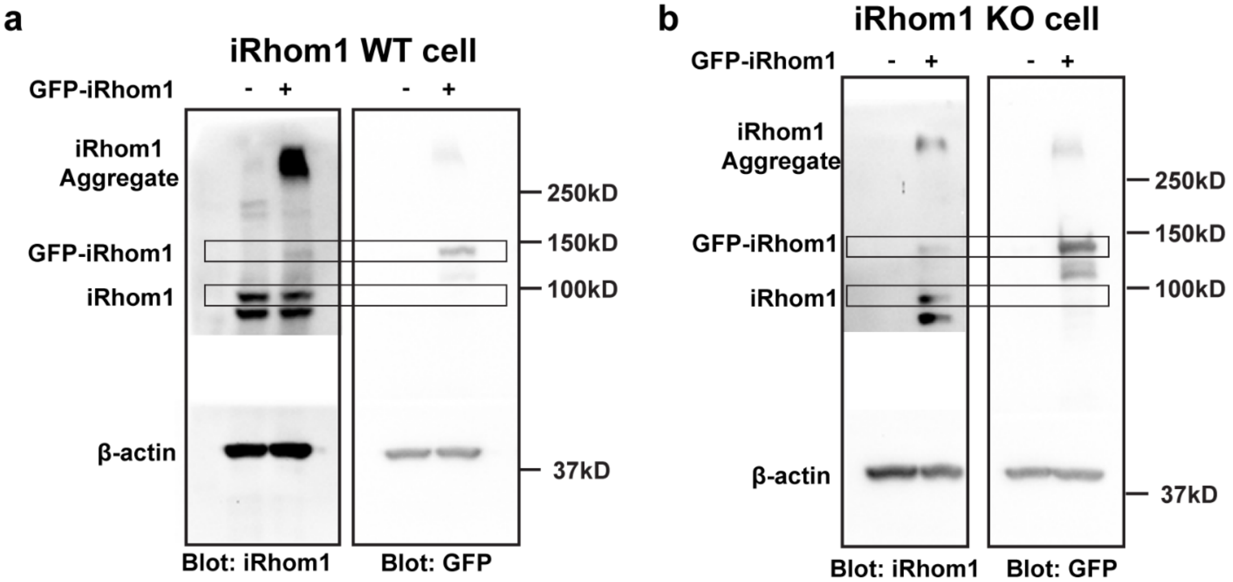

Supplementary Figure 4. Characterization of iRhom1 overexpression in CT26 cells **(a)** and iRhom1 rescue in CT26 iRhom1$^{-/-}$ cells **(b)**. Data are representative of two independent experiments in a, b. Source data are provided as a Source Data file for all panels.

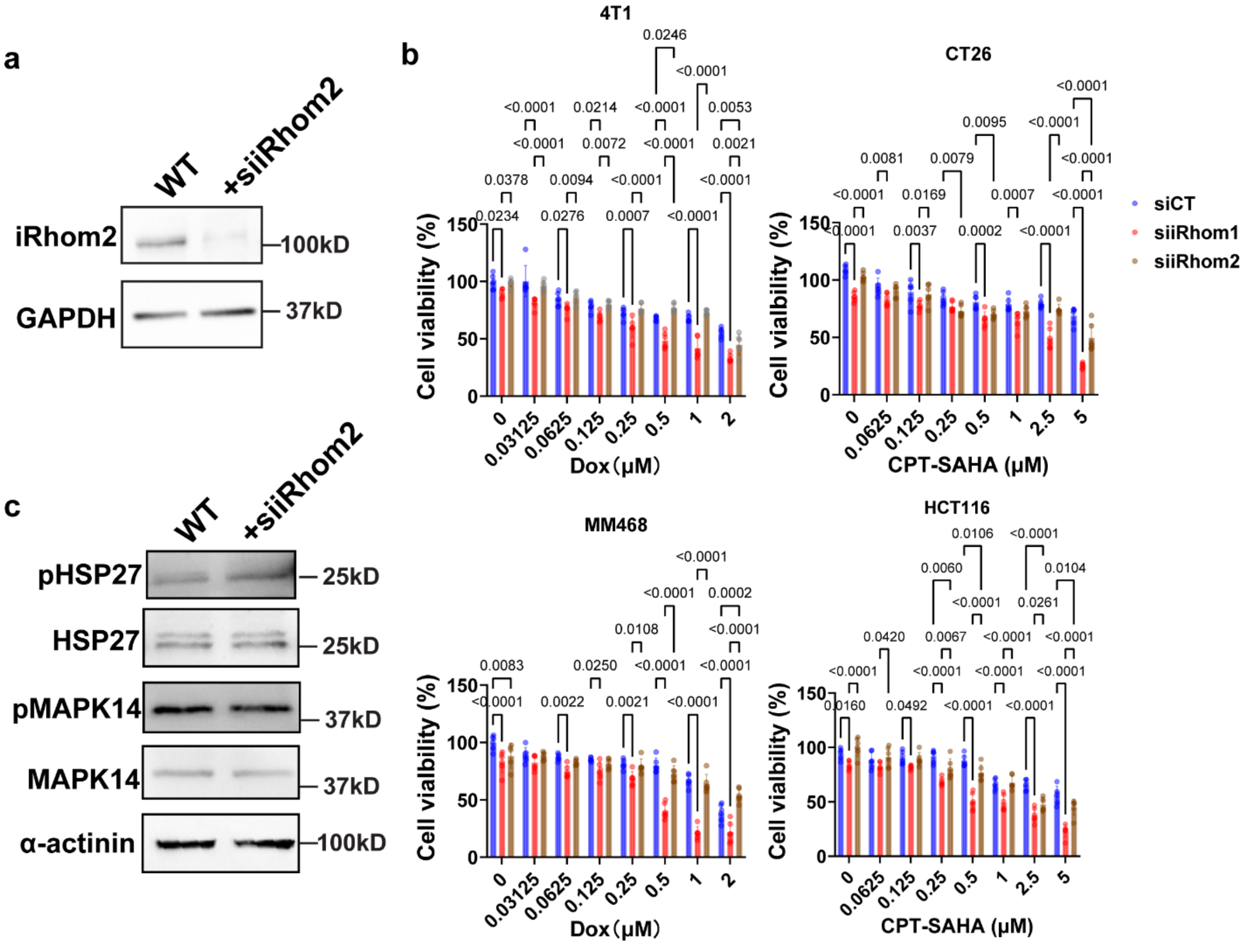

Supplementary Figure 5. (a) Validation of iRhom2 KD after siiRhom2 treatment. **(b)** The impact of siRNA-mediated knockdown of iRhom1 or iRhom2 on the cytotoxicity of DOX or CPT-SAHA in several cancer cell lines (MTT assay). N = 6 independent samples. **(c)** IRhom2 KD showed no significant impact on the protein levels of MAPK14-HSP27 axis. Data are presented as mean ± s.e.m. in b. Statistical analysis was performed by one-way ANOVA with Tukey's post hoc test for comparison in b. Data are representative of two independent experiments in a, c, and three independent experiments in b. Source data are provided as a Source Data file for all panels.

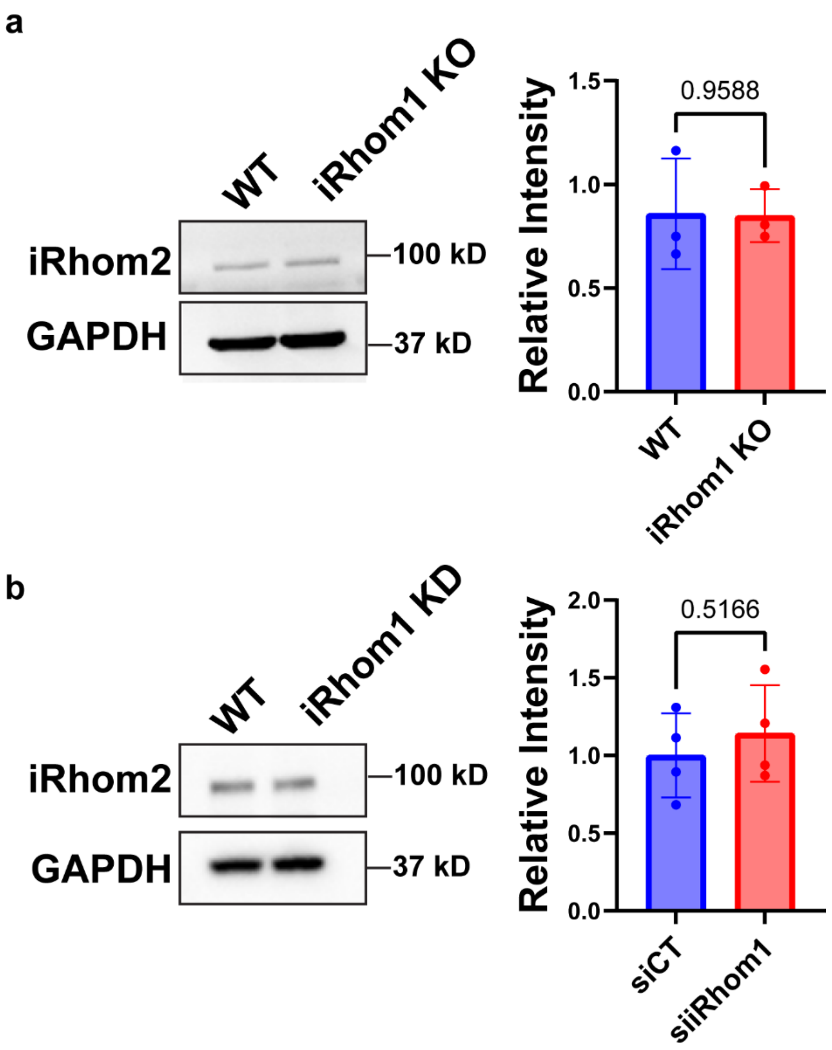

Supplementary Figure 6. (a) Changes in protein levels of iRhom2 in iRhom1 KO cells. N=3 independent experiments. **(b)** Changes in protein levels of iRhom2 in iRhom1 KD cells. N=4 independent experiments. Data are presented as mean ± s.e.m. in a and b. Statistical analysis was performed by two-tailed Student's t-test for comparison in a and b. Source data are provided as a Source Data file for all panels.

.

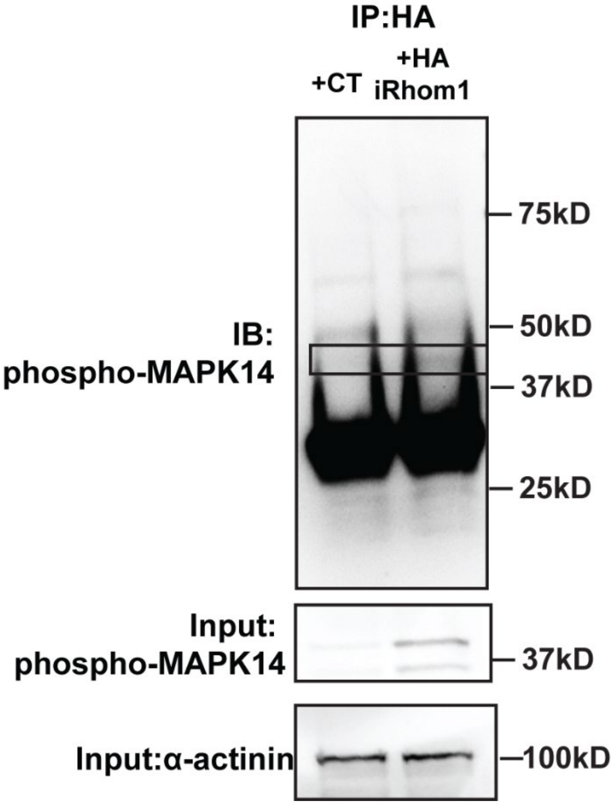

Supplementary Figure 7. Immunoprecipitation of HA-iRhom1 with anti-HA beads led to pulldown of phospho-p-38α in 293T cells transfected with a HA-iRhom1 expression plasmid. Data are representative of two independent experiments. Source data are provided as a Source Data file.

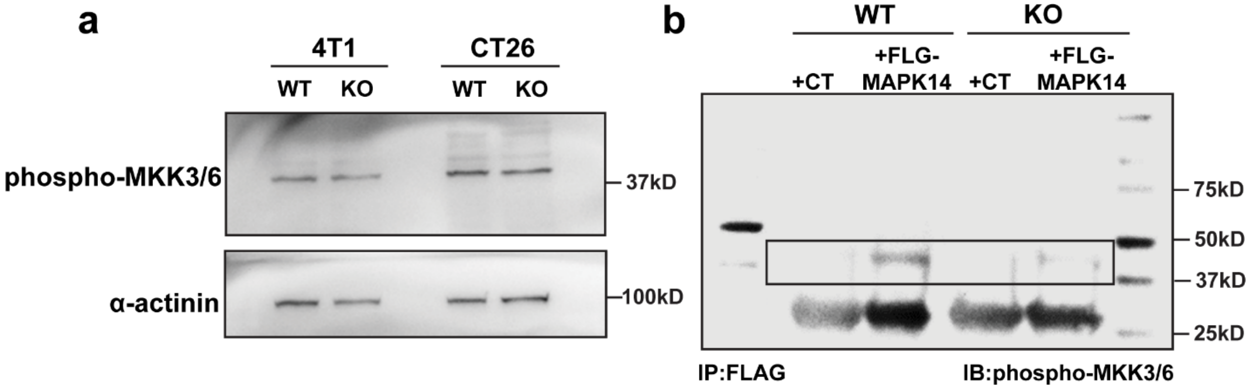

Supplementary Figure 8. (a) IRhom1 KO shows no impact on the protein levels of p-MKK3/6 in 4T1 or CT26 cells. **(b)** Immunoprecipitation of FLAG-MAPK14 with anti-FLAG antibody led to pulldown p-MKK3/6 in iRhom1 WT but not in KO cells transfected with a FLAG-MAPK14 expression plasmid. Data are representative of two independent experiments in a, b. Source data are provided as a Source Data file for all panels.

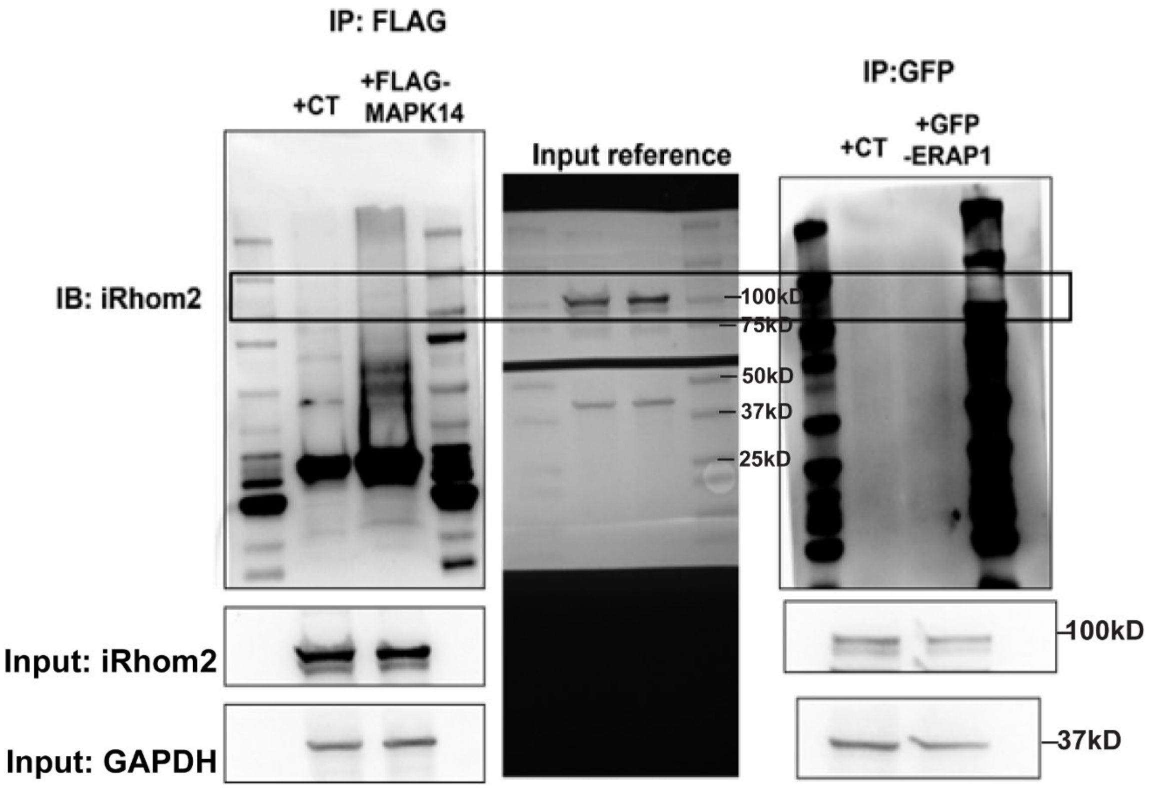

Supplementary Figure 9. Immunoprecipitation of FLAG-MAPK14 with anti-FLAG beads or GFP-ERAP1 with anti-GFP beads failed to pulldown iRhom2 in 293T cells transfected with a FLAG-MAPK14 or GFP-ERAP1 expression plasmid respectively. Data are representative of two independent experiments. Source data are provided as a Source Data file for all panels.

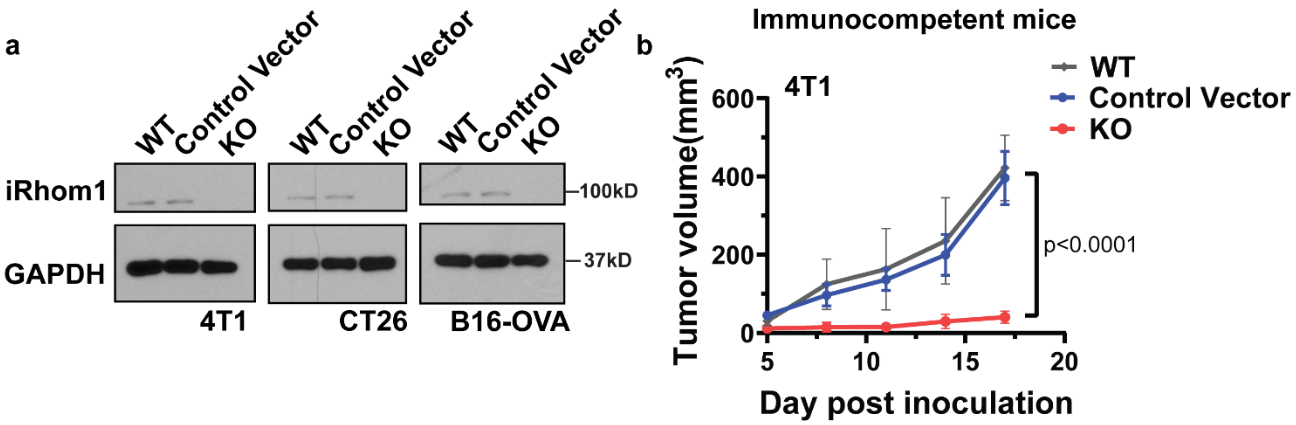

Supplementary Figure 10. (a) Characterization of iRhom1 KO CT26, 4T1 and B16-OVA cell lines. **(b)** Tumor growth curves of WT cells, control vector cells, and iRhom1 KO 4T1 cells on immunocompetent mice. N=5 animals. Data are presented as mean ± s.e.m. in b. Statistical analysis was performed by one-way ANOVA with Tukey's post hoc test for comparison in b. Data are representative of two independent experiments in a, b. Source data are provided as a Source Data file for all panels.

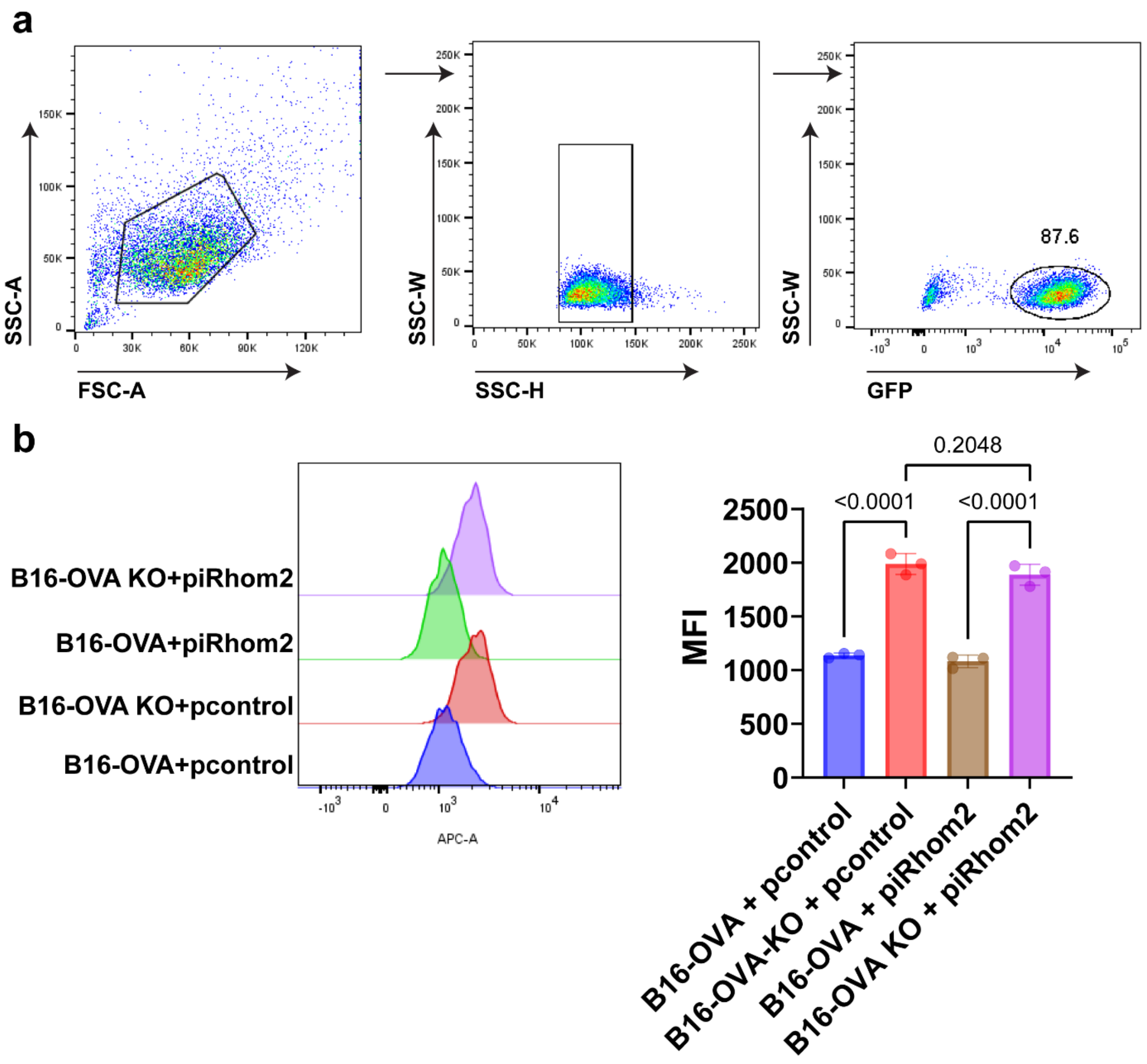

Supplementary Figure 11. (a) Gating strategy of GFP^+ cells in pGFP-iRhom2 transfected iRhom1 KO B16-OVA cells. **(b)** Histograms and the quantification of H2Kb/SIINFEKL expression on WT or iRhom1 KO B16-OVA cells following iRhom2 transgene expression. N=3 independent samples. Data are presented as mean ± s.e.m. in b. Statistical analysis was performed by one-way ANOVA with Tukey's post hoc test for comparison in b. Data are representative of two independent experiments. Source data are provided as a Source Data file for b.

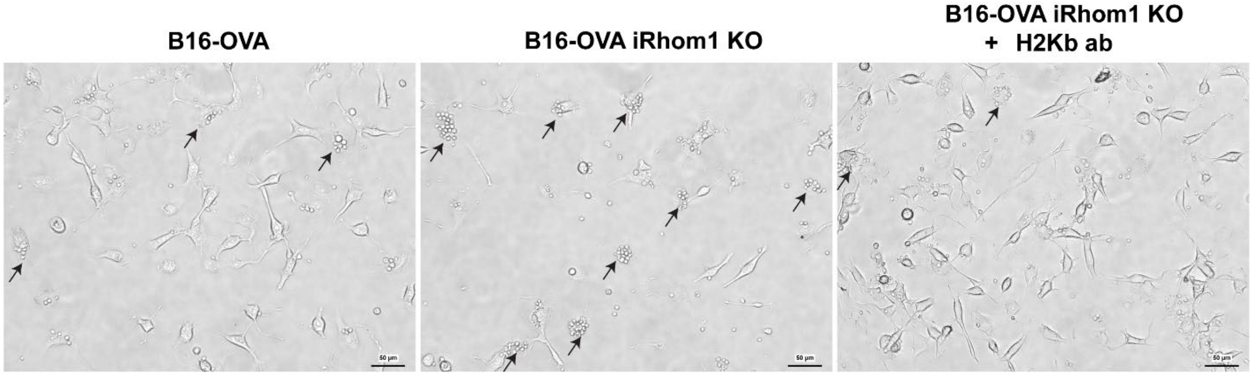

Supplementary Figure 12. Microscopic images of the co-culture showing killing of tumor cells by CD8$^+$ T cells. Scale bar, 50 μm. Data are representative of two independent experiments.

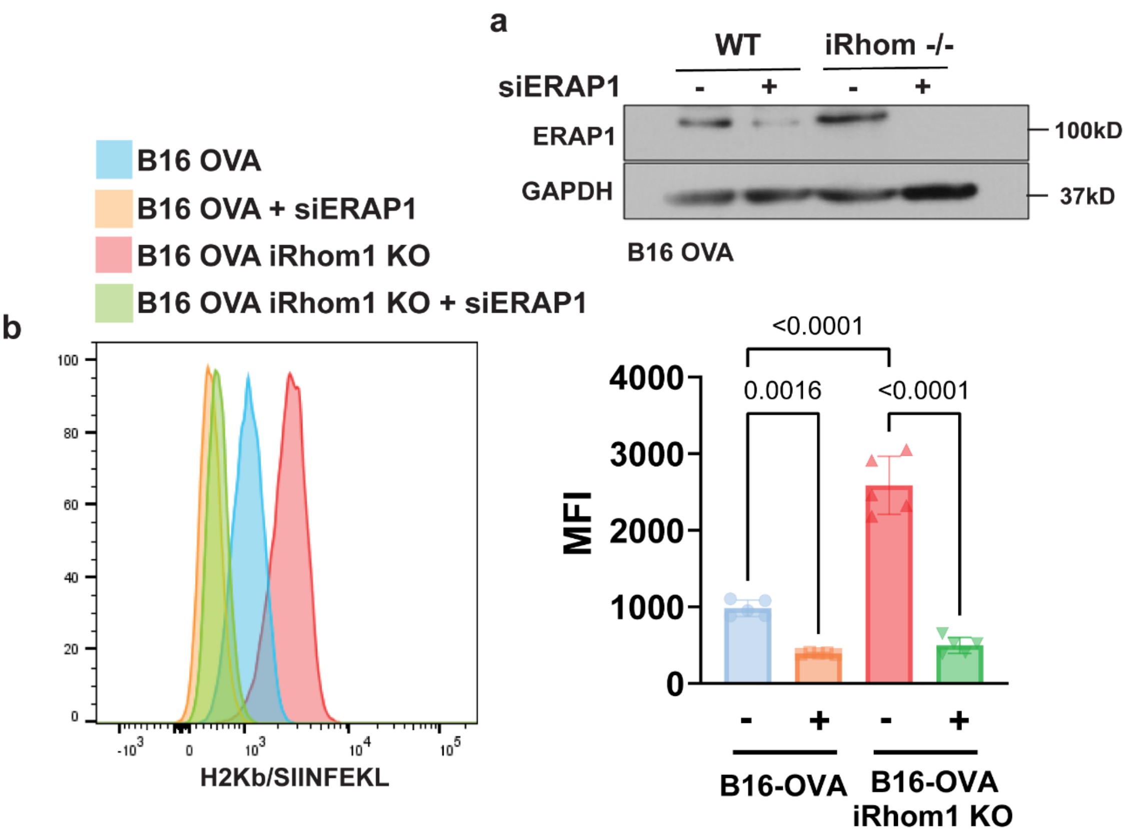

Supplementary Figure 13. (a) Characterization of ERAP1 knockdown in WT or iRhom1 KO B16-OVA cells. **(b)** Histograms and the quantification of H2Kb/SIINFEKL expression on B16-OVA WT or B16-OVA iRhom1 KO cells following ERAP1 knockdown. N=5 independent samples. Data are presented as mean ± s.e.m. in b. Statistical analysis was performed by one-way ANOVA with Tukey's post hoc test for comparison in b. Data are representative of two independent experiments in a, b. Source data are provided as a Source Data file for all panels.

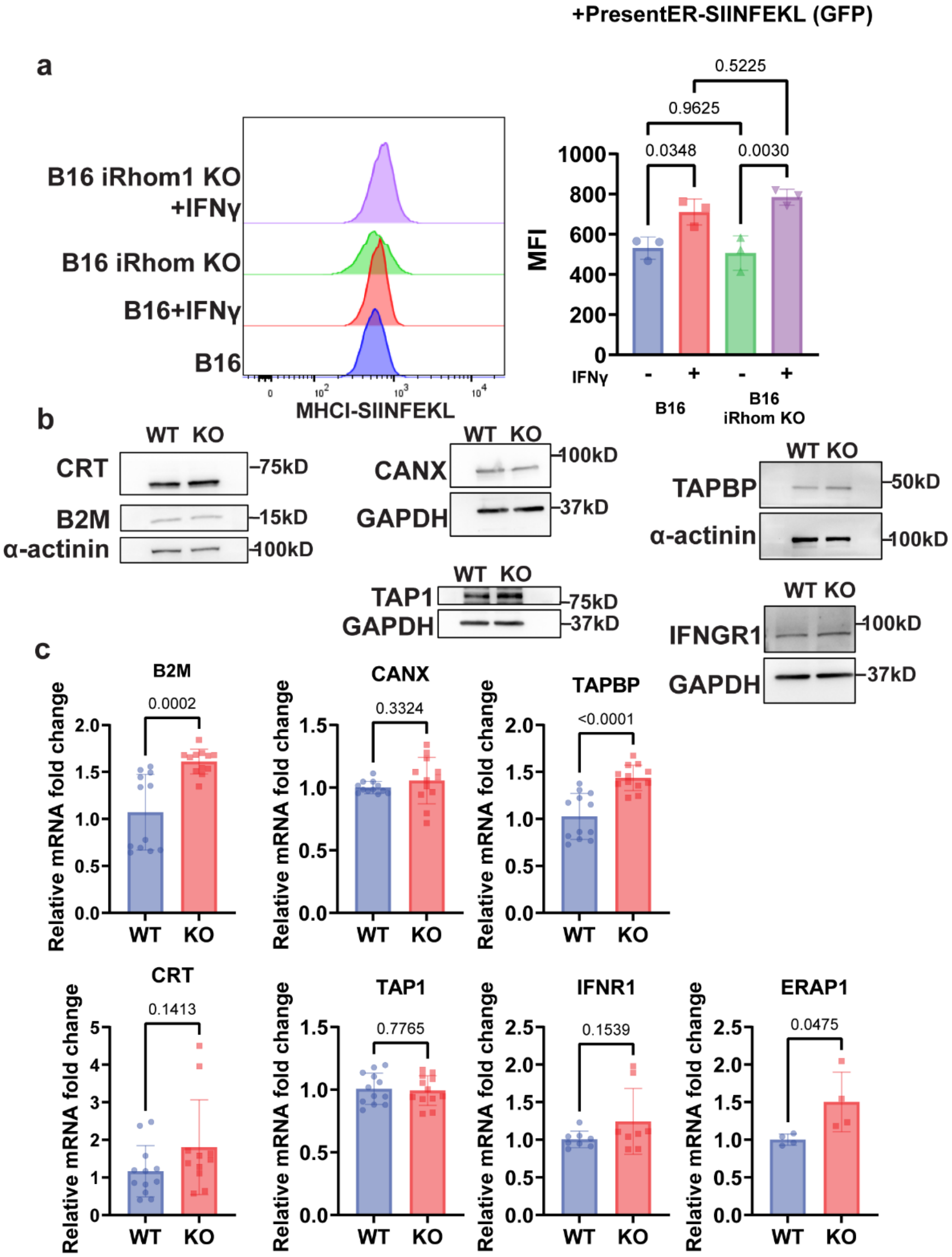

Supplementary Figure 14. (a) Histograms and the quantification of H2KB/SIINFEKL expression on B16 WT or B16 iRhom1 KO cells that were transduced with a virus expressing PresentER minigene encoding H2-Kb ligand SIINFEKL. N=3 independent samples. **(b-c)** The impact of iRhom1 KO on the expression levels of several components in APP at protein (**b**) and mRNA **(c)** levels. N=12 independent samples in B2M, CRT, CANX, TAP1, TAPBP. N=8 independent samples in IFNGR1. N=4 independent samples in ERAP1. B2M: beta-2 macroglobulin; CRT:

calreticulin; CANX: calnexin; TAP1: antigen peptide transporter 1; TAPBP: TAP binding protein; IFNGR1: interferon-gamma receptor 1; ERAP1: endoplasmic reticulum aminopeptidase 1. Data are presented as mean ± s.e.m. in a, c. Statistical analysis was performed by one-way ANOVA with Tukey's post hoc test for comparison in a, and two-tailed Student's t-test for comparison in c. Data are representative of two independent experiments in a, b and three independent experiments in c. Source data are provided as a Source Data file for all panels.

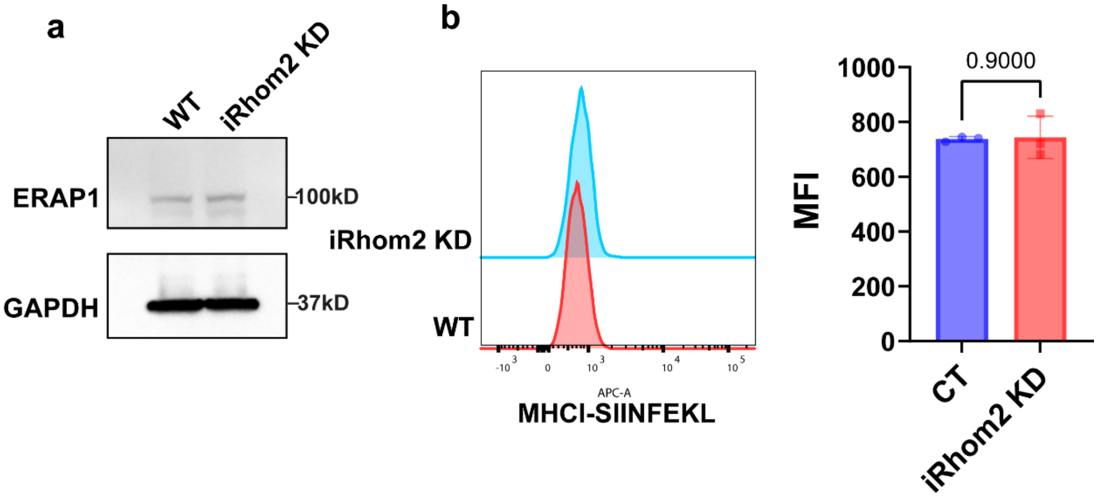

Supplementary Figure 15. (**a**) KD of iRhom2 shows no effect on the protein levels of ERAP1 in CT26 cells. (**b**) Histograms and the quantification of H2KB/SIINFEKL expression on B16-OVA WT or iRhom2 KD cells. N=3 independent samples. Data are presented as mean ± s.e.m. in b. Statistical analysis was performed by two-tailed Student's t-test for comparison in b. Data are representative of two independent experiments in a, b. Source data are provided as a Source Data file for all panels.

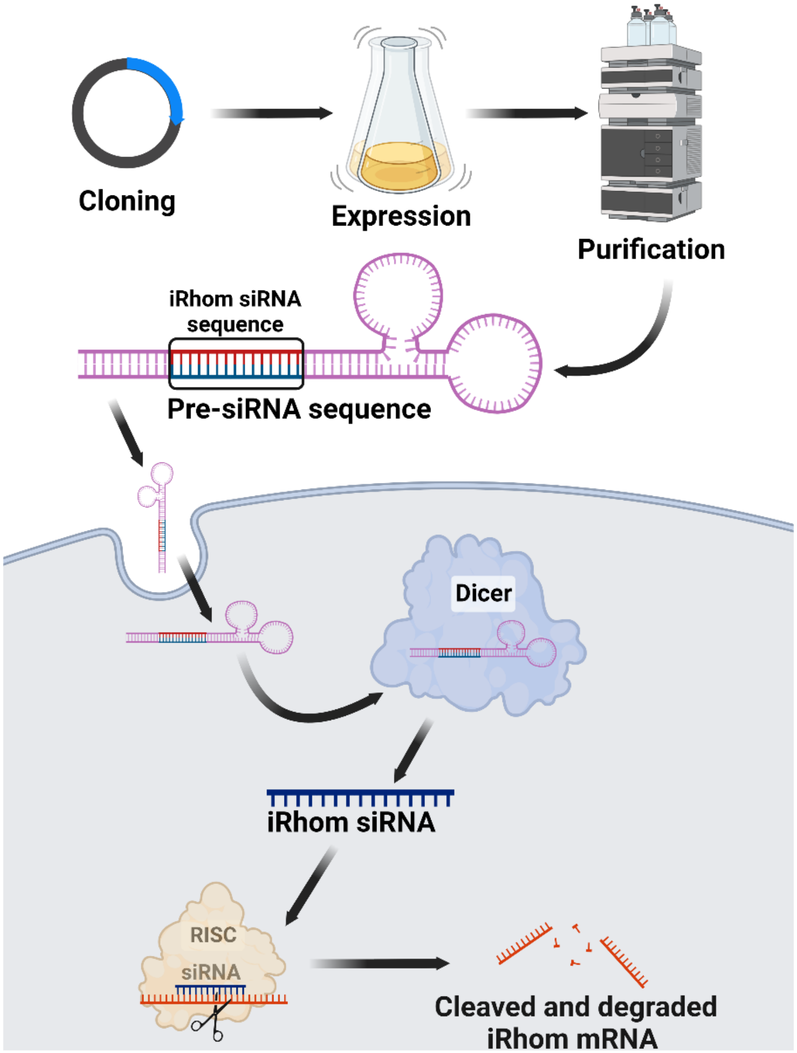

Supplementary Figure 16. RNA bioengineering platform technology for generation of iRhom1 pre-siRNA. After a target pre-siRNA is designed and its expression plasmid is cloned, expression of target RNA in bacteria is readily determined by RNA gel electrophoresis. Target RNAs are purified with proper methods (e.g., anion exchange FPLC). Pre-siRNA will be processed by dicer to generate mature siRNA intracellularly, and lead to targeted mRNA degradation. The figure is created with BioRender.com.

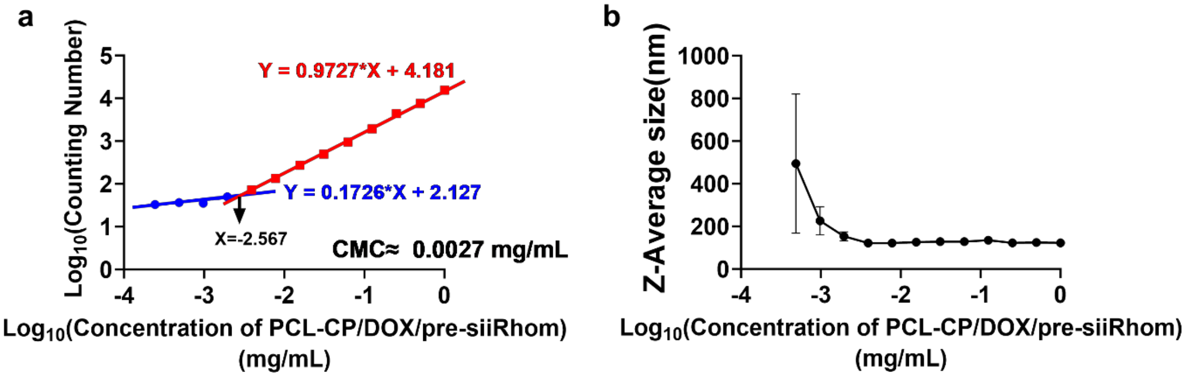

Supplementary Figure 17. (a) Critical micelle concentration of DOX/pre-siiRhom1 PCL-CP construct. **(b)** Changes in size of DOX/pre-siiRhom1 PCL-CP at different dilutions. N=3 independent samples. Data are presented as mean ± s.e.m. Data are representative of two independent experiments in a, b. Source data are provided as a Source Data file for all panels.

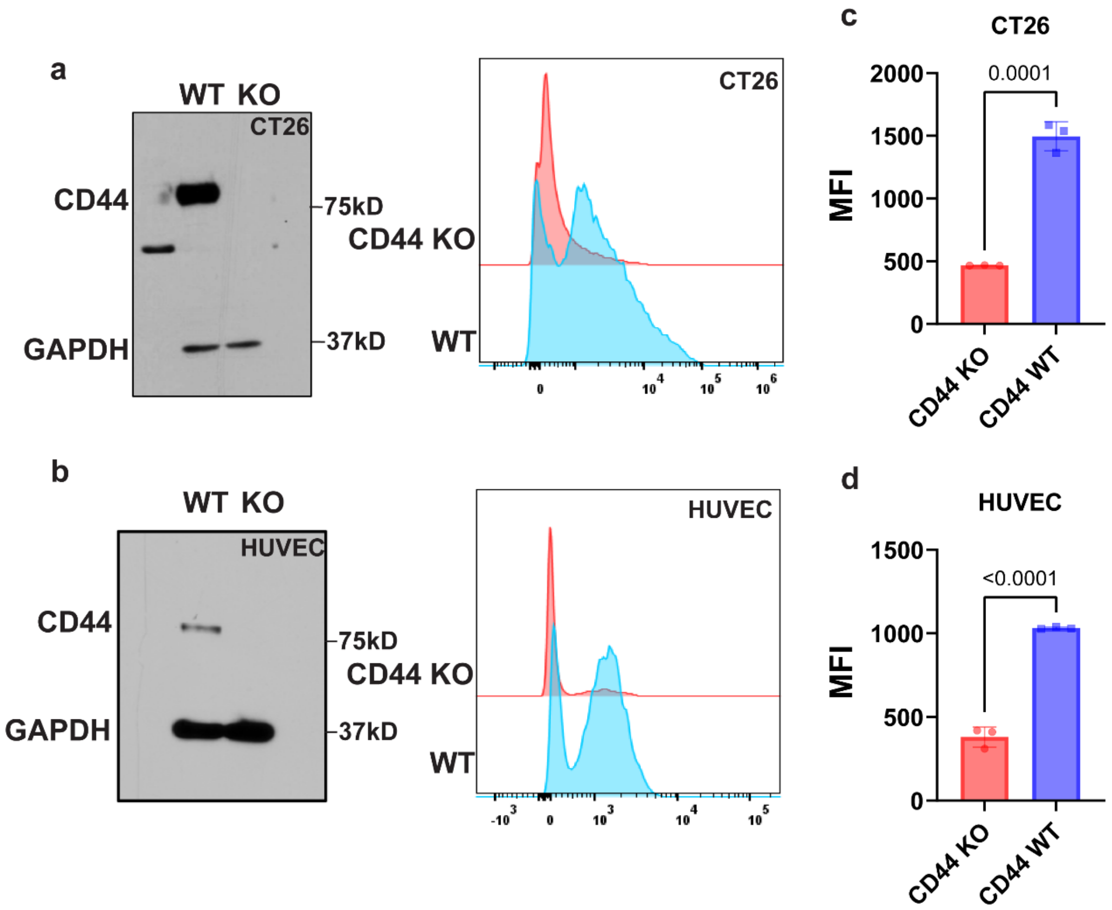

Supplementary Figure 18. (a) Characterization of CD44 KO CT26 tumor cells by Western blot. (**b**) Characterization of CD44 KO HUVEC cells by Western blot. **(c)** Cellular uptake of Cy5.5 labeled PCL-CP NPs in WT or CD44 KO CT26 cells. N=3 independent samples. (**d**) Cellular uptake of Cy5.5 labeled PCL-CP NPs in WT or CD44 KO HUVEC cells. N=3 independent samples. Data are presented as mean ± s.e.m. in c, d. Statistical analysis was performed by two-tailed Student's t-test for comparison in c, d. Data are representative of two independent experiments in a-d. Source data are provided as a Source Data file for all panels.

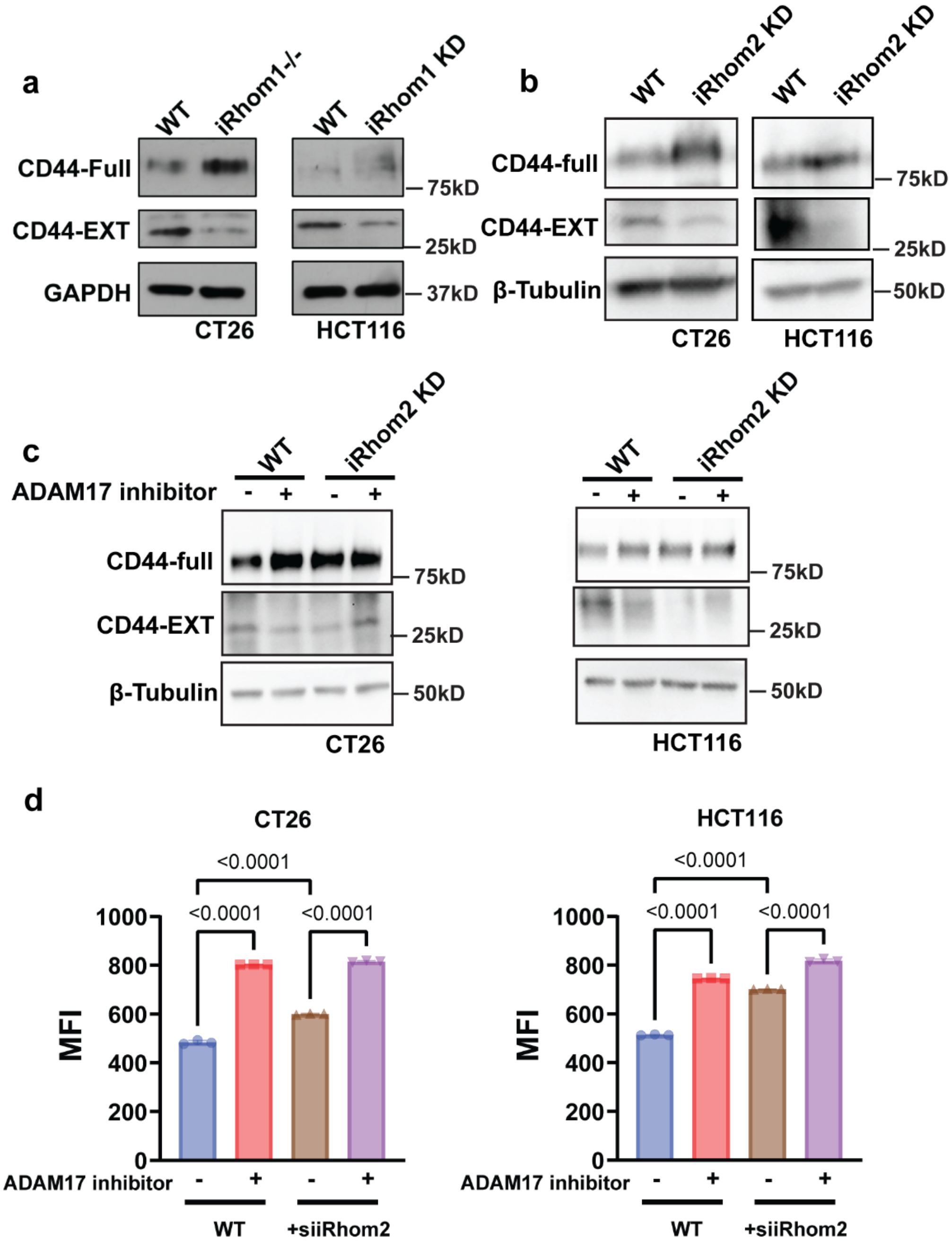

Supplementary Figure 19. (a) Changes in protein levels of the full-length CD44 (CD44-Full) and the cleaved membrane-bound fragment of CD44 (CD44-EXT) in WT or iRhom1 knockout/knockdown cells (blotted by an antibody recognizing the CD44 intracellular domain). **(b)** Changes in protein levels of the full-length CD44 (CD44-Full) and the cleaved membrane-bound fragment of CD44 (CD44-EXT) in WT or iRhom2 knockdown cells. **(c)** Changes in protein levels of full-length CD44 (CD44-Full) and the cleaved, CD44 membrane-bound fragment (CD44-EXT) in WT or iRhom2 knockdown cells, with or without ADAM17 inhibitor pretratment. **(d)** Cellular uptake of Cy5.5 labeled PCL-CS NPs in WT or iRhom2 knockdown cells with or without ADAM17 inhibitor pretreatment. N=3 indepdent samples. Data are presented as mean ± s.e.m. in d. Statistical analysis was performed by one-way ANOVA with Tukey's post hoc test for comparison

in d. Data are representative of two independent experiments in a-d. Source data are provided as a Source Data file for all panels.

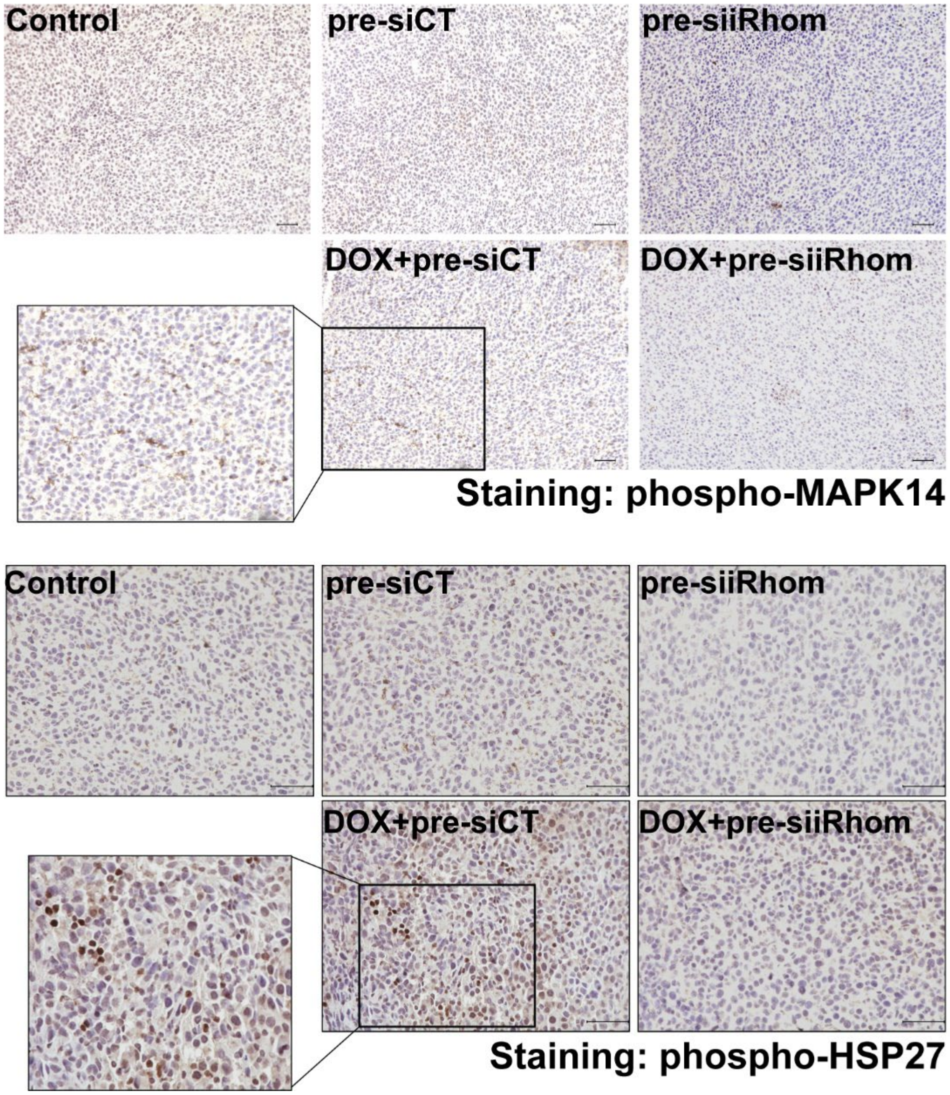

Supplementary Figure 20. IHC staining of phospho-p38a and phospho-HSP27 in tumor tissues after various treatments (Bar=100 nm). Data are representative of two independent experiments.

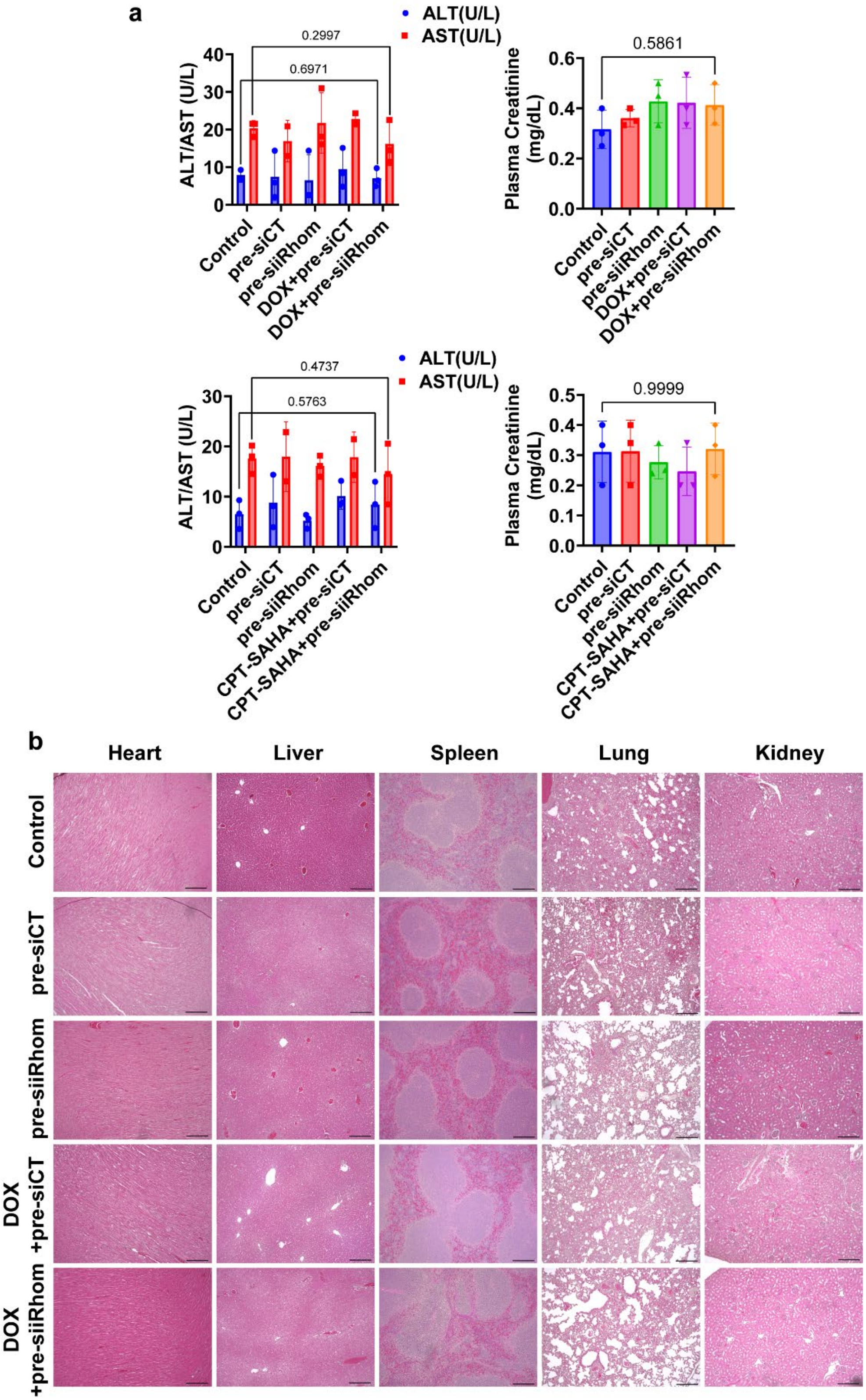

Supplementary Figure 21. (a) Serum levels of ALT, AST, and creatinine after various treatments. N=3 independent samples. **(b)** H&E staining of major organs after various treatments. Bar=200 μm. Data are presented as mean ± s.e.m. in a. Statistical analysis was performed by one-way

ANOVA with Tukey's post hoc test for comparison in a. Data are representative of two independent experiments in a, b. Source data are provided as a Source Data file for a.

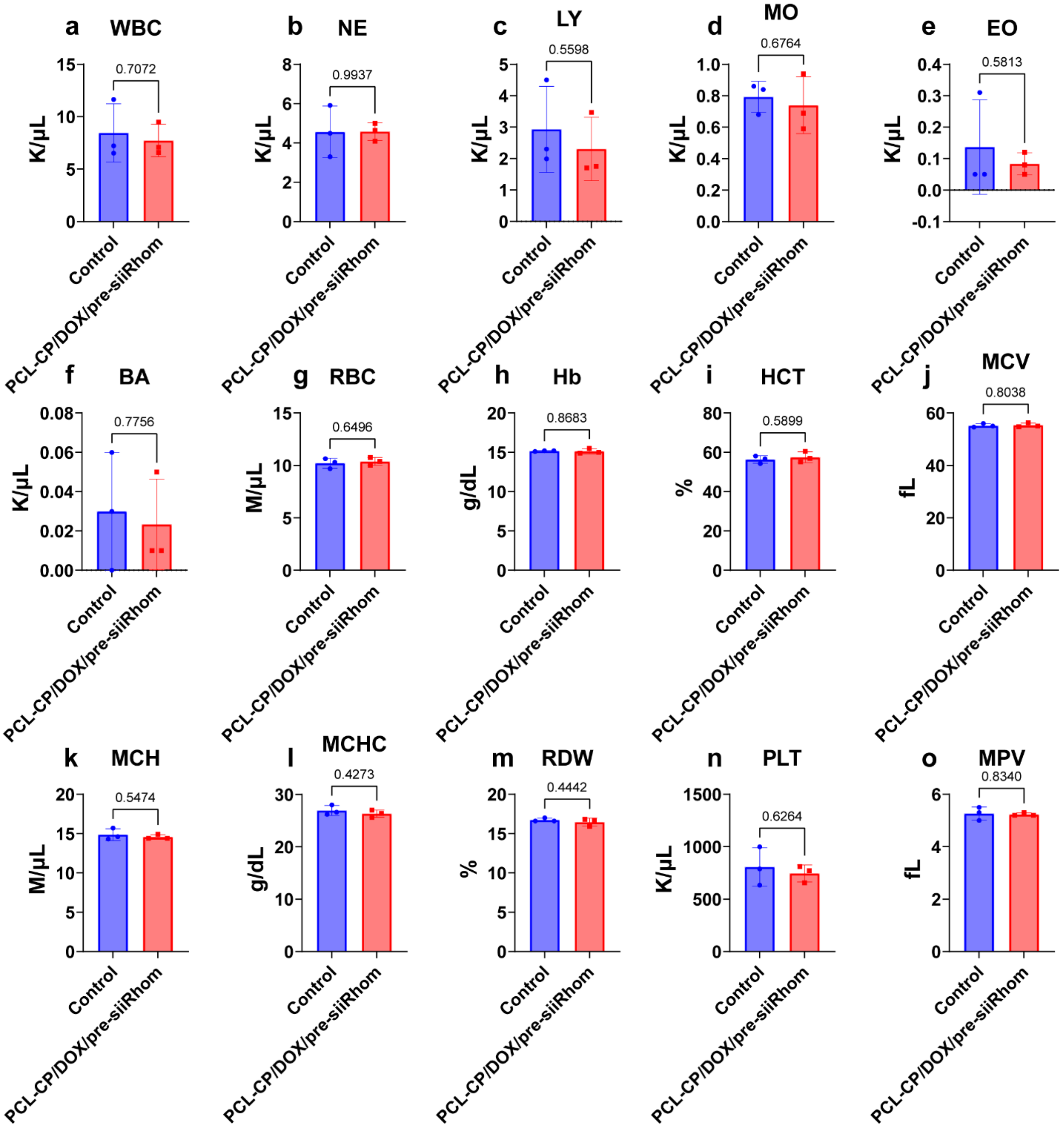

Supplementary Figure 22. (a-o) Changes in various parameters of blood cells after various treatments. N=3 independent samples. Data are presented as mean ± s.e.m. in all panels. Statistical analysis was performed by two-tailed Student's t-test for comparison in all panels. Data are representative of two independent experiments in a-o. Source data are provided as a Source Data file for all panels.

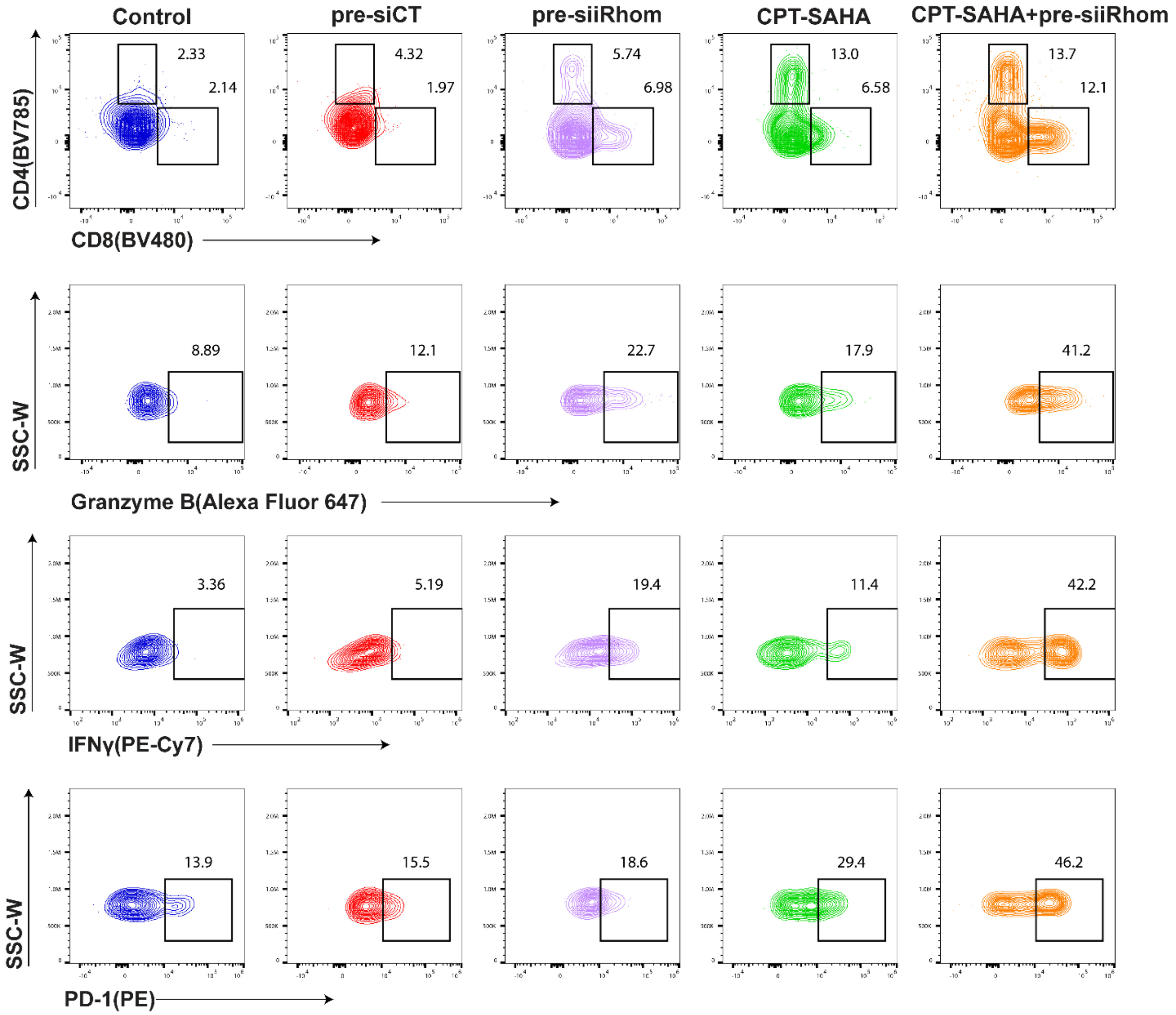

Supplementary Figure 23. Representative flow cytometric analysis of the relative abundance of $CD8^+$ T-cells, $CD4^+$ T-cells, CD8+ $IFN\gamma^+$ T-cells, $CD8^+$ Granzyme B+ T-cells and $CD8^+$ PD1+ T-cells in CT26 tumor tissues after various treatments.

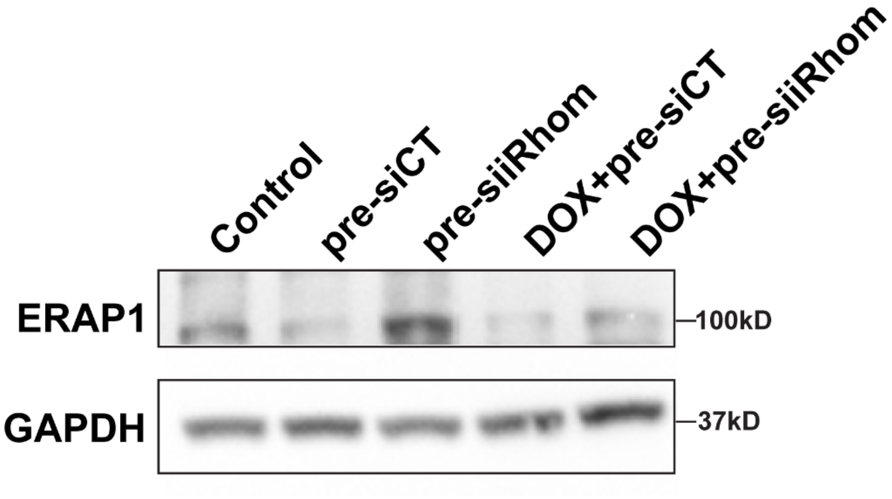

Supplementary Figure 24. Changes in ERAP1 protein levels in tumors after various treatments. Data are representative of two independent experiments. Source data are provided as a Source Data file.

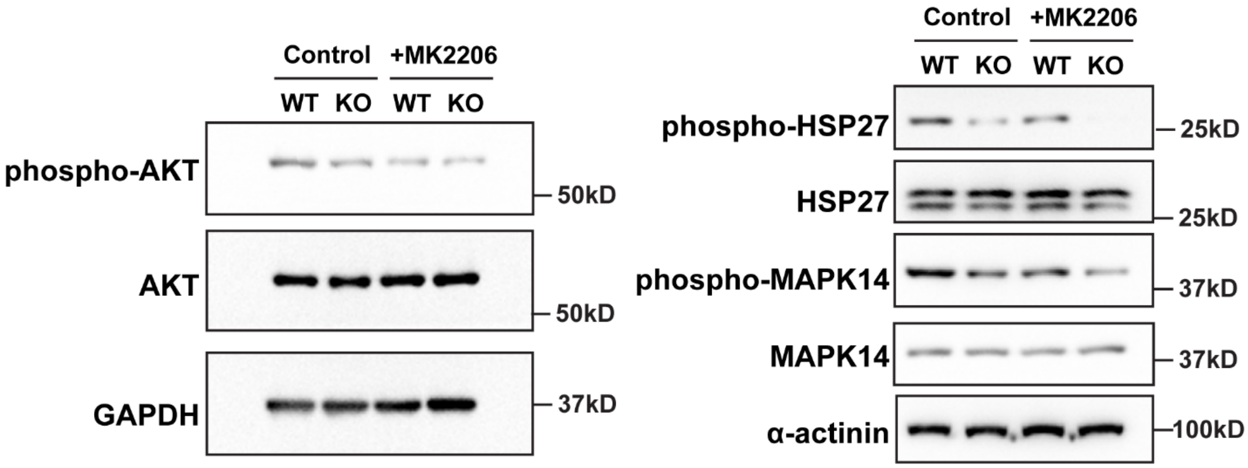

Supplementary Figure 25. Changes in MAPK-HSP27 after inhibition of PI3K-AKT pathway by AKT inhibitor MK2206 in WT and iRhom1 KO CT26 cells. Data are representative of two independent experiments. Source data are provided as a Source Data file for all panels.

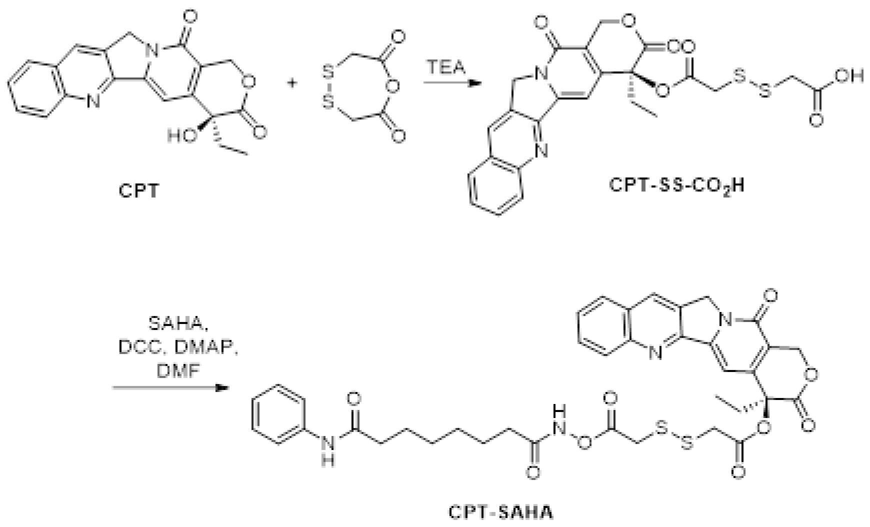

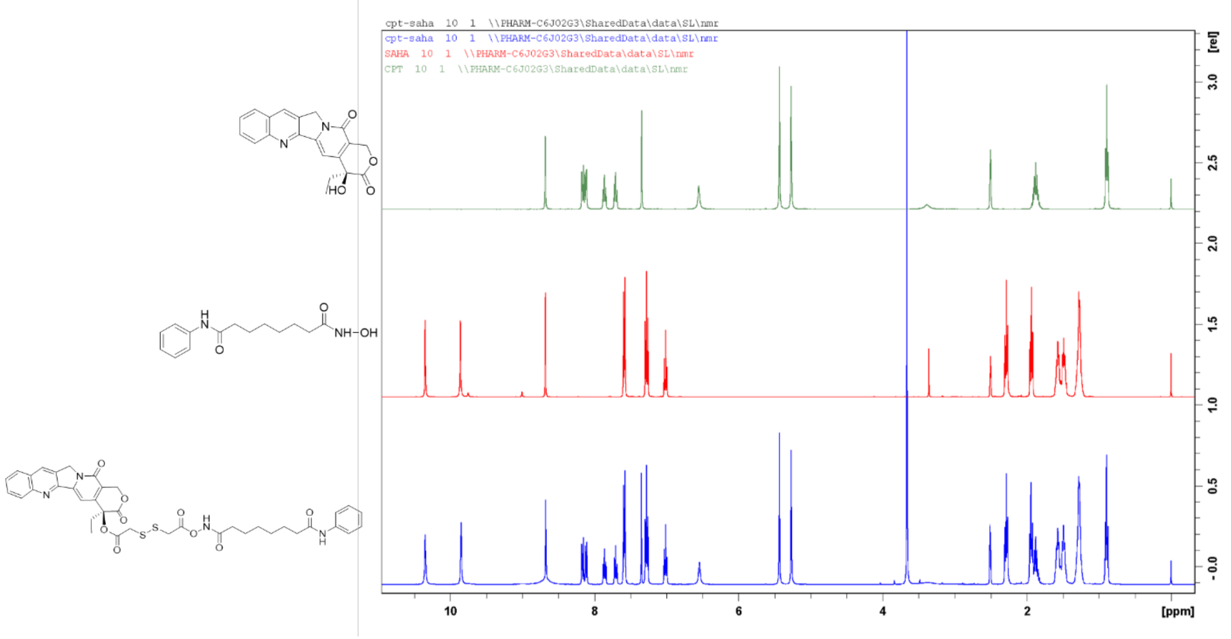

Supplementary Figure 26. Synthesis route and ^{1}H-NMR (in DMSO-*d*6) of CPT-SAHA. NMR of SAHA and campthothecin were used as reference. 1H NMR (400 MHz, DMSO-*d*6): δ 10.35 (s, 1H), 9.85 (s, 1H), 8.68 (s, 1H), 8.16 (d, *J* = 8.5 Hz, 1H), 8.11 (d, J = 8.12, 1H), 7.86 (t, J = 7.4 Hz, 1H), 7.71 (t, J = 7.8 Hz, 1H), 7.58 (d, J = 8 Hz, 2H), 7.35 (s, 1H), 7.27(t, J = 7.8 Hz, 3H), 7.01 (t, J = 7.4 Hz, 1H), 5.44(s, 1H), 5.27(s, 1H), 3.67 (s, 4H), 2.28 (t, J = 7.4Hz, 3H), 1.88-1.96(m, 5H), 1.47-1.57 (m, 6H), 1.26-1.29 (m, 6H), 0.90 (t, J = 7.2 Hz, 3H).

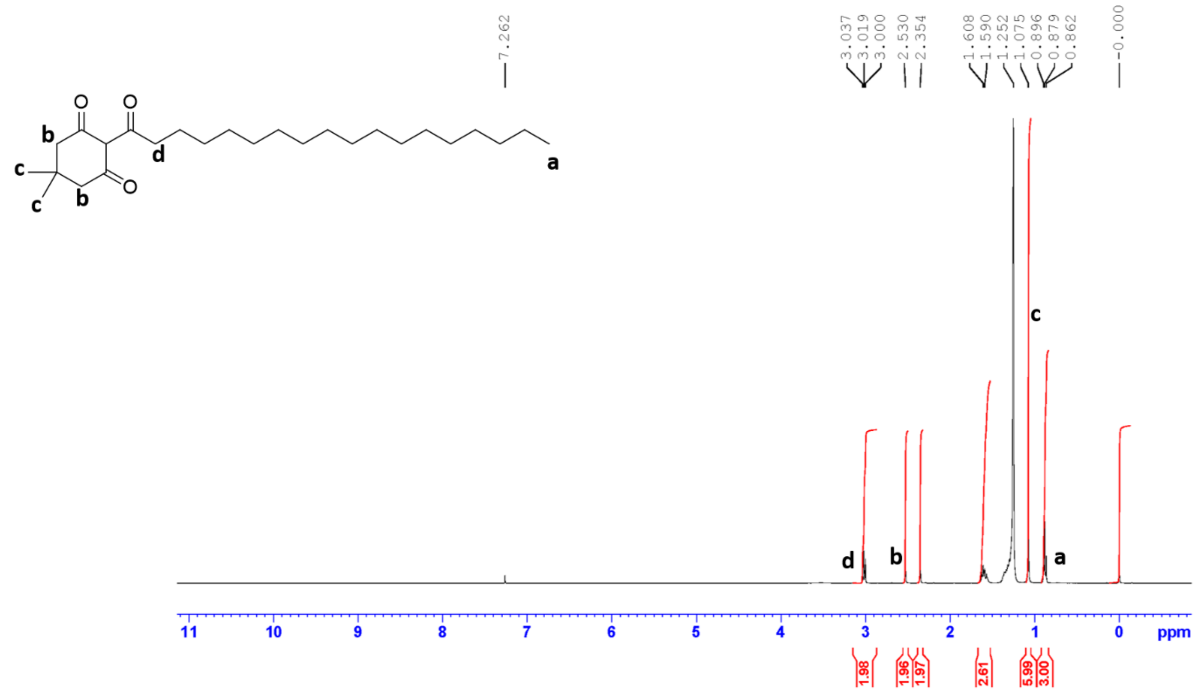

Supplementary Figure 27. ^{1}H-NMR (in $CDCl_3$-*d*) of 2-stearoylcyclohexane-1,3-dione. Each peak with label represents the feature structure. 1H NMR (400 MHz, CDCl3): δ 3.02 (t, *J* = 7.6 Hz, 2H), 2.54(s, 2H), 2.35(s, 2H), 1.60 (m, 2H), 1.25 (m, 30 H), 1.08 (s, 6H), 0.88 (t, J = 6.8 Hz, 3H).

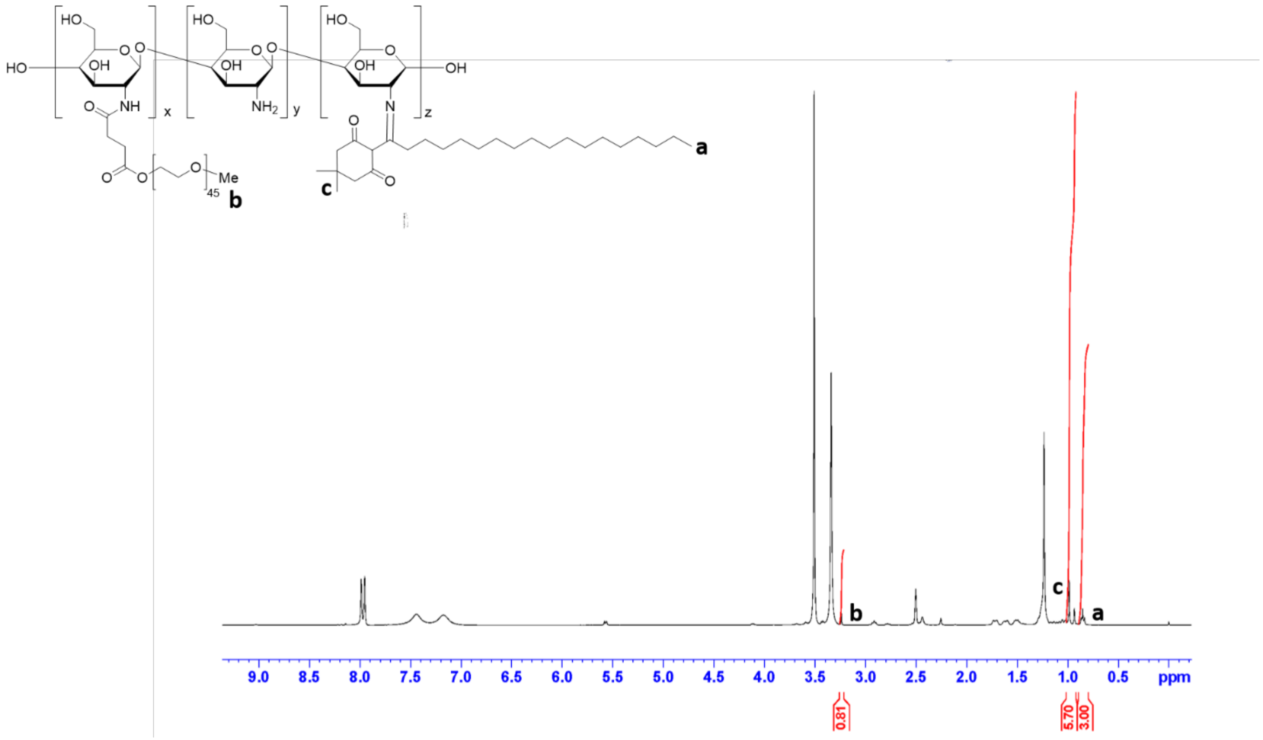

Supplementary Figure 28. ^{1}H-NMR (in DMSO-*d*6) of PEG-Chitosan-lipid (PCL) with Schiff base linker. Each peak with label represents the feature structure of the PCL polymer. Peak a is methyl group (δ 0.85, t, J = 3.24 Hz, 3H). Peak b is PEG's terminal methoxy group (δ 3.24, s, 3H). Peak c is di-methyl group (δ 0.98, s, 6H). PEG: lipid=1:4, calculated based on the integration ratio between peak a and peak b.

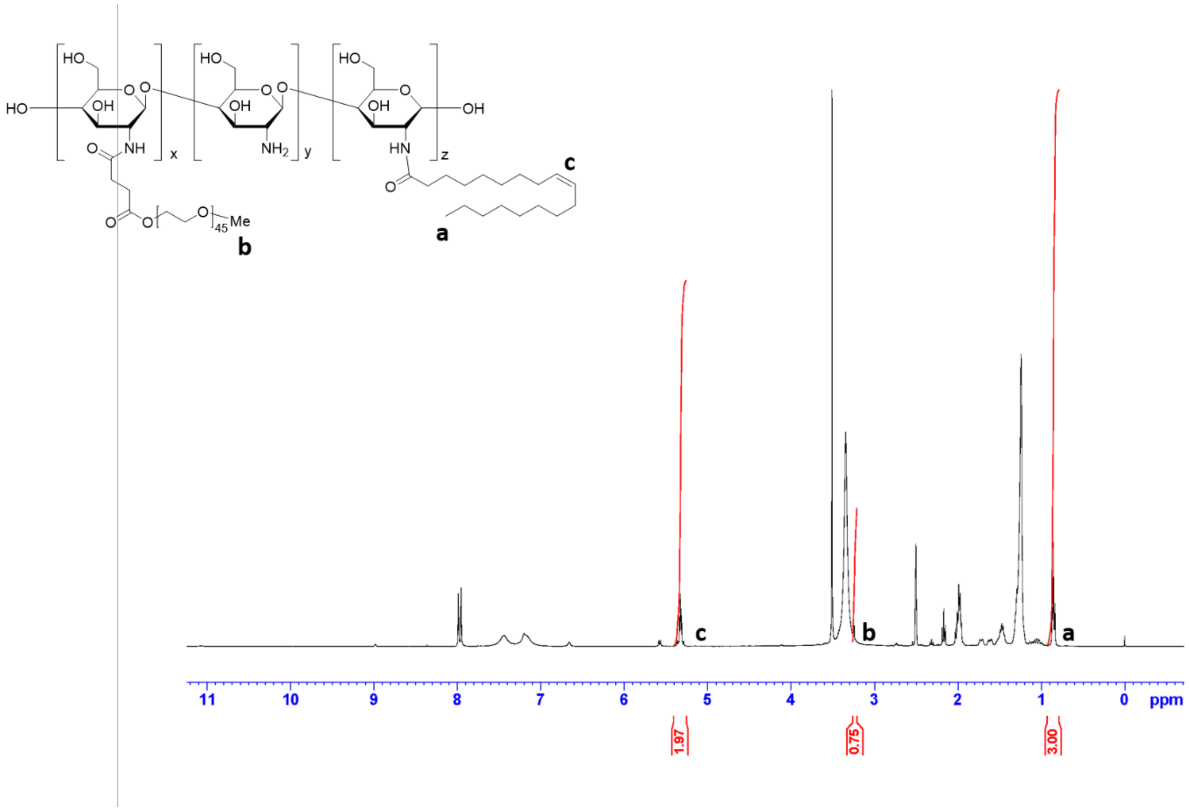

Supplementary Figure 29. 1H-NMR (in DMSO-*d*6) of PEG-Chitosan-lipid (PCL) with amide linker. Each peak with label represents the feature structure of the PCL polymer. Peak a is methyl group (δ 0.85, t, J = 3.24 Hz, 3H). Peak b is PEG's terminal methoxy group (δ 3.24, s, 3H). Peak c is olefinic hydrogen of oleyl acid motif (δ 5.32, m, 2H). PEG: Lipid=1:4, calculated based on the integration ratio between peak a and peak b.

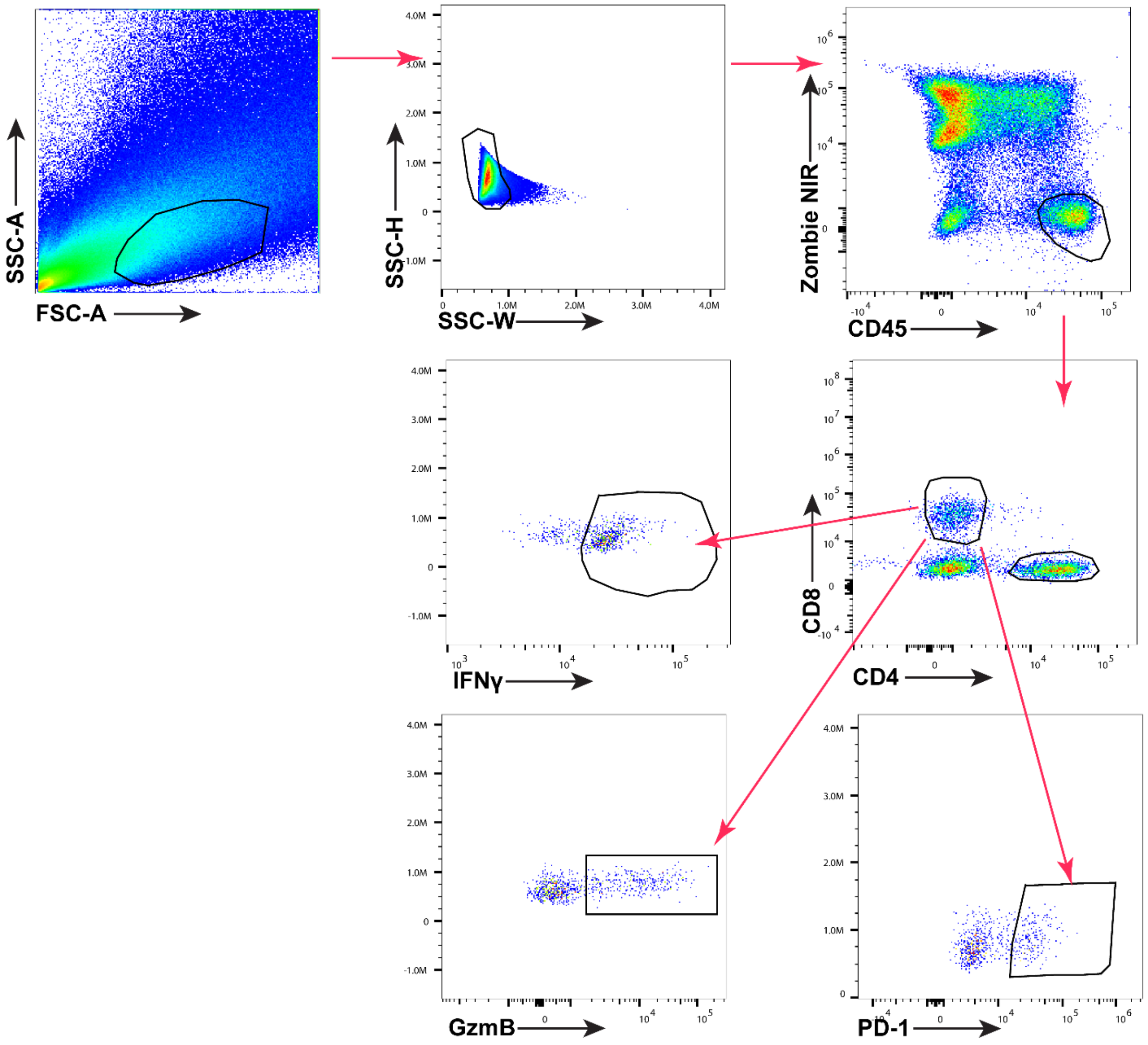

Supplementary Figure 30. Gating strategies of figure 7 for analysis of CD45⁺ cells, CD4⁺ T cells, CD8⁺ T cells, IFNγ⁺, GzmB⁺ and PD-1⁺ CD8⁺ cells in tumor tissues.

Supplementary Table 1. Antibody list

Producer	Cat. No	Lot No	Dilution
Invitrogen	PA5-110292	WF3296625	1/1000
Abcam	ab81342	GR3274561-2	1/1000
Cell signaling technology	2401	14	1/500
Cell signaling technology	2242	2	1/1000
Sigma Aldrich	09-272	DAM1503386	1/500
Cell signaling technology	9218	7	1/1000
Cell signaling technology	43603	6	1/2000
Cell signaling technology	4695	28	1/1000
Cell signaling technology	4370	3	1/1000
Cell signaling technology	4691	4	1/1000
Cell signaling technology	4060	4	1/1000
Cell signaling technology	9126	6	1/1000
Cell signaling technology	9154	22	1/1000
Abcam	ab124669	GR3240239-2	1/1000
Abcam	ab157107	GR3391745-11	1/500
Cell signaling technology	4695	9	1/2000
Biorbyt	orb386934	AC4964	1/1000
Cell signaling technology	12238	4	1/1000
Cell signaling technology	66382	17	1/1000
Proteintech	13511-1-AP	78730	1/1000
Proteintech	11114-1-AP	18281	1/1000
Proteintech	10427-2-AP	10026770	1/1000
Proteintech	10808-1-AP	00049501	1/1000
Cell signaling technology	2956	8	1/2000
Cell signaling technology	4970	11	1/2000
Cell signaling technology	2146S	7	1/2000
Cell signaling technology	6487	4	1/2000
Cell Signaling Technology	7074S	31	1/5000
Cell Signaling Technology	5127	18	1/5000
Abcam	ab222783	GR3300671-2	1/50
Cell Signaling Technology	39037	3	1/100
Novus Biologicals	NB500-138	QC07519-15732	1/100

Cell Signaling Technology	9079	7	1/100
BD Biosciences	561862	1334565	1/200
eBioscience	17-5979-82	2410170	1/200
Biolegend	141605	B273749	1/200
Biolegend	423105	B328963	1/1000
Biolegend	30-F11	B280746	1/200
Biolegend	100453	B354442	1/200
BD Biosciences	566096	1165100	1/200
BD Biosciences	557649	1243760	1/200
Biolegend	515405	B301363	1/200
BD Biosciences	561788	1337063	1/200
Biolegend	124308	B278302	1/200
Bioxcell	BP0033-2	778521M1	100µg per mouse
Bioxcell	BE0077	736121S1	0.5 µg for 5 × 10^4 CD8+ T cells

Supplementary Table 2. Oligonucleotide sequence

Primer for qPCR		
Name	Forward Sequence (5'-3')	Reverse Sequence (5'-3')
m-Rhbdf2	GCCCACACCGTATCTGTTCTG	GATGCCAGTTTTGTCGCTTGC
m-Erap1	CCTGTCTGAGAGTTTCCATG	ATCAAAGCAGGGAAAAGCCA
m-Ifngr1	CCTGTCAGAGGTGTCCCTCG	GGGAGACCTTAGGACAGCTC
m-B2m	AATAAATGAAGGCGGTCCCAGG	TGGTGCCCTACTATCTAGGGTG
m-Tap1	GAGAAGAACACGACAGGCCA	TCAGGCTGTTCTGGAAGCTG
m-Tapbp	TCCCAACACCCCTCTGTTTG	CGCCACCTCCCTTAAAACCA
m-Crt	GGAAGATGAGGAGGAAGATGTC	CAGGAAGGAGAGCAGATGAAAT
m-Canx	GTGGTGCCTATGTGAAGCTGCT	GCAGTTTGTAGTCCTCTCCACAC

Primer for pre-siRNA quantification	
Stem-Loop RT primer	GTCGTATCCAGTGCAGGGTCCGAGGTATTCGCACTGGATACGACACATCA
qPCR forward	CACGCAGAGTGAGCAAGG
qPCR Reverse	CCAGTGCAGGGTCCGAGGTA

Pre-siiRhom sequence (5’-3’)
ACCAGGAUGGCCGAGUGGUUAAGGCGUUGGACUGGCCAGCUGUGAGUGUUUCUUGA GUGAGCAAGGACAGUGAUGUUGUGAGCAAUAGUAAGGAAGCAUCACUGCCUCUGCUC ACUAUAGAAGUGCUGCACGUUGUUGGCCCGAUCCAAUGGACAUAUGUCCGCGUGGGU UCGAACCCCACUCCUGGUACCA
