# Supplementary material for: Inhibition of iRhom1 by CD44-targeting nanocarrier for improved cancer immunochemotherapy
Source: Nat Commun. 2024 Jan 4;15:255. doi: 10.1038/s41467-023-44572-6 (PMC10766965; doi:10.1038/s41467-023-44572-6)
Supplement: Supplementary file 3 — Description of Additional Supplementary Files [file 41467_2023_44572_MOESM3_ESM.pdf]

Description of Additional Supplementary Files

Supplementary Data 1: full list of clustered drugs based on pharmacological targets and their average correlation Z score.

Supplementary Data 2: full list of compounds that exhibit higher drug sensitivity with lower iRhom1 expression.
